# Neuroprotective Effect of Glatiramer Acetate on Neurofilament Light Chain Leakage and Glutamate Excess in an Animal Model of Multiple Sclerosis

**DOI:** 10.3390/ijms222413419

**Published:** 2021-12-14

**Authors:** Rina Aharoni, Raya Eilam, Shaul Lerner, Efrat Shavit-Stein, Amir Dori, Joab Chapman, Ruth Arnon

**Affiliations:** 1 Department of Immunology, The Weizmann Institute of Science, Rehovot 761001, Israel; ruth.arnon@weizmann.ac.il; 2 Department of Brain Sciences, The Weizmann Institute of Science, Rehovot 761001, Israel; raya.eilam@weizmann.ac.il; 3 Department of Biological Regulation, The Weizmann Institute of Science, Rehovot 761001, Israel; shaulerner@gmail.com; 4 Department of Neurology, Sheba Medical Center, Ramat Gan 5262000, Israel; efrat.shavit.stein@gmail.com (E.S.-S.); amir.dori@gmail.com (A.D.); joabchapman@gmail.com (J.C.); 5 Department of Neurology and Neurosurgery, Sackler Faculty of Medicine, Tel Aviv University, Tel Aviv 6997801, Israel; 6 Robert and Martha Harden Chair in Mental and Neurological Diseases, Sackler Faculty of Medicine, Tel Aviv University, Tel Aviv 6997801, Israel

**Keywords:** multiple sclerosis (MS), experimental autoimmune encephalomyelitis (EAE), glatiramer acetate (GA), neurodegeneration, neurofilament light (NFL), glutamate, neuroprotection

## Abstract

Axonal and neuronal pathologies are a central constituent of multiple sclerosis (MS) and its animal model, experimental autoimmune encephalomyelitis (EAE), induced by the myelin oligodendrocyte glycoprotein (MOG) 35–55 peptide. In this study, we investigated neurodegenerative manifestations in chronic MOG 35–55 induced EAE and the effect of glatiramer acetate (GA) treatment on these manifestations. We report that the neuronal loss seen in this model is not attributed to apoptotic neuronal cell death. In EAE-affected mice, axonal damage prevails from the early disease phase, as revealed by analysis of neurofilament light (NFL) leakage into the sera along the disease duration, as well as by immunohistological examination. Elevation of interstitial glutamate concentrations measured in the cerebrospinal fluid (CSF) implies that glutamate excess plays a role in the damage processes inflicted by this disease. GA applied as a therapeutic regimen to mice with apparent clinical symptoms significantly reduces the pathological manifestations, namely apoptotic cell death, NFL leakage, histological tissue damage, and glutamate excess, thus corroborating the neuroprotective consequences of this treatment.

## 1. Introduction

In multiple sclerosis (MS) and its animal model, experimental autoimmune encephalomyelitis (EAE), the immune system (including T-cells, B-cells, and components of the innate immune system) reacts against the myelin envelope that surrounds the axons, resulting in demyelination and tissue damage [1,2,3]. Axonal and neuronal pathology is a central constituent of MS, manifested from the disease onset by swelling and transection of axons and loss of neurons, leading to an irreversible clinical disability and disease progression [2,3,4,5,6,7,8]. Consequently, MS is increasingly acknowledged as a neurodegenerative disease triggered by an inflammatory attack on the CNS [6,7,8]. While both inflammation and demyelination are well recognized, the processes involved in neurodegeneration are less defined. However, it is accepted that neurodegeneration comprises a combination of neuronal cell death, apoptosis, necrosis, and hypoxia [8].

We have previously demonstrated that chronic EAE induced by the myelin oligodendrocyte glycoprotein (MOG) 35–55 peptide in C57BL/6 mice is particularly characterized by various neuronal and axonal pathologies. Thus, immunohistochemical and ultrastructural analysis in brains and spinal cords revealed in this model transections and deformations of axons, as well as swelling of neuronal cell bodies and nucleus margination [9,10]. Furthermore, quantitative analysis throughout the L4–L5 ventral horn uncovered a progressive reduction in motor neurons in EAE-inflicted mice, reaching 46% neuronal loss by day 50 after disease induction [9]. The degeneration prevailing in this MOG-induced model was also evident using diffusion tensor imaging (DTI), showing a significant increase in the apparent diffusion coefficient (ADC), indicative of structural CNS damage [11]. The ultimate consequence of the degenerative process, cognitive deterioration, was manifested in this model by impairments in working and long-term memory, starting at the early stages and increasing with disease progression [12]. These characteristics are either absent or less prominent in other EAE models, such as the relapsing remitting EAE model induced by the myelin proteolipid protein (PLP) 139–151 peptide in SJL/J mice [9,11].

The crucial role of neuroaxonal damage in determining the clinical outcome emphasizes the need of reliable biomarkers for in situ quantification of neurodegeneration, in an attempt to improve disease activity assessment and treatment outcome. Neurofilaments (NFs) comprise a tissue-specific class of cytoskeletal intermediate filaments located exclusively in neurons [13]. In the adult CNS they appear as heteropolymers of four subunits: neurofilament light (NFL), neurofilament middle (NF-M), neurofilament heavy (NF-H), and α-internexin. As components of the cytoskeleton, NFs provide structural support and form a regionally specialized network that assembles proteins and organelles. Disruptions of NFs expression, organization, or metabolism are associated with various neurodegenerative and neuropsychiatric disorders [13,14]. Furthermore, reduced NFs expression is a general response to axonal injury [9,15]. Since NFs proteins are released from damaged axons, their elevated levels in the cerebrospinal fluid (CSF) or the serum have been used as biomarkers for axonal/neuronal injury in various pathological situations [13,14,16]. In MS particularly, the presence (“leakage”) of the NFL subunit and its quantitative fluctuation in the serum/plasma emerges as a promising prognostic indicator to monitor neuro-axonal damage and disease progression [17,18,19]. Many studies have recently used the ultrasensitive single molecule array (Simoa) technology for NFL quantification in MS patients, to assess disease activity and treatment consequence, revealing strong correlations between serum NFL levels, MRI parameters and relapse rate [17,18,19,20,21,22]. In animal models of MS a few studies demonstrating NFL measurements were also reported [23,24]. Yet, the limited understanding of NFL kinetics in the sera presents a barrier for its usage as a standard biomarker.

Several mechanisms have been implicated in contributing to the neurodegeneration in the course of MS and EAE. These include chronic microglial and astrocytic activation, altered expression of ion channels, mitochondrial damage, oxidative stress, and iron accumulation [8,25,26]. Such processes, in combination with the characteristic burst of pro-inflammatory cytokines, such as TNF-α and IL-1β, are known to affect the local level of glutamate [27,28,29,30]. This major excitatory neurotransmitter, secreted in the CNS by multiple cell types, including the invading immune cells, induces when in excess excitotoxic cell death of neurons and loss of brain function, thus constituting a therapeutic target [27,28,29,30,31]. In MS and EAE, multiple abnormalities in glutamate degrading enzymes, transporters, receptors and signaling have been reported [27,28,29,30,31], but measuring the actual glutamate concentration is less employed due to the irrelevance of its serum level and the difficulty in assessing it’s brain interstitial concentration.

The currently used disease-modifying therapies (DMT) have only partially limited neurodegeneration [32,33]. Therefore, it is essential to further test their effect on the characteristic axonal and neuronal pathologies. Glatiramer acetate (GA, Copaxone) is a first-line DMT used worldwide in patients with relapsing remitting MS. The mechanism of action of GA is mainly attributed to immunomodulation, competing with myelin antigen for binding to MHC class II molecules, and inducing deviation from pro-inflammatory T helper (Th)-1 and Th-17 to anti-inflammatory Th2 and T-regulatory pathways [34,35]. A growing number of findings indicate that GA treatment also leads to augmentation of neuroprotective processes, such as elevation of neurotrophic factors including brain derived neurotrophic factor (BDNF) in EAE-mice as well as in MS patients [35,36,37]. Utilizing immunohistochemistry and electron microscopy in the EAE model, we observed protective outcome of GA on the disease’s primary target, the myelin [9,38,39]. Notably, reduced axonal damage as well as reduced neuronal loss are evident in the CNS of GA-treated mice compared to EAE-untreated mice [9,10,11]. Furthermore, GA augments the proliferation of neuronal progenitor cells, which diverge from the classic migratory streams and spread to damage sites in brain regions that do not normally undergo neurogenesis [10]. The typical cognitive deterioration shown in MOG-induced EAE-mice is also prevented by GA [12]. Findings from human studies support the notion that GA reduces the neuropathological damage in MS patients. Thus, GA treatment reduced the formation of permanent T1 hypo-intense lesions that evolve into “black holes”, associated with irreversible neurological disability [40]. It was also shown that GA treatment leads to a significant increase in the NAA:Cr ratio compared to pre-treatment values, implying an axonal metabolic recovery and protection from sub-lethal axonal injury [41]. These cumulative findings support the notion that GA-treatment augments neuroprotective processes and counteracts the neurodegenerative disease-pathologies.

In this study, we aimed to further investigate the neurodegenerative manifestations in the MOG-induced EAE model and the effect of GA-treatment on these manifestations. We report herewith that in contrast to previous claims the neuronal loss seen in this chronic model cannot be attributed to apoptotic neuronal cell death. In EAE-inflicted mice, early onset of axonal damage prevails, as revealed by a detailed kinetics of NFL leakage into the sera along the disease duration and by immunohistological analysis. Elevation of interstitial glutamate concentrations indicates that glutamate excess contributes to the damage inflicted on the CNS cell populations in this disease. A therapeutic regimen of GA given to mice with apparent clinical symptoms significantly reduced NFL leakage, tissue damage, as well as glutamate excess, thus corroborating its neuroprotective effect.

## 2. Results

**Figure 1:**
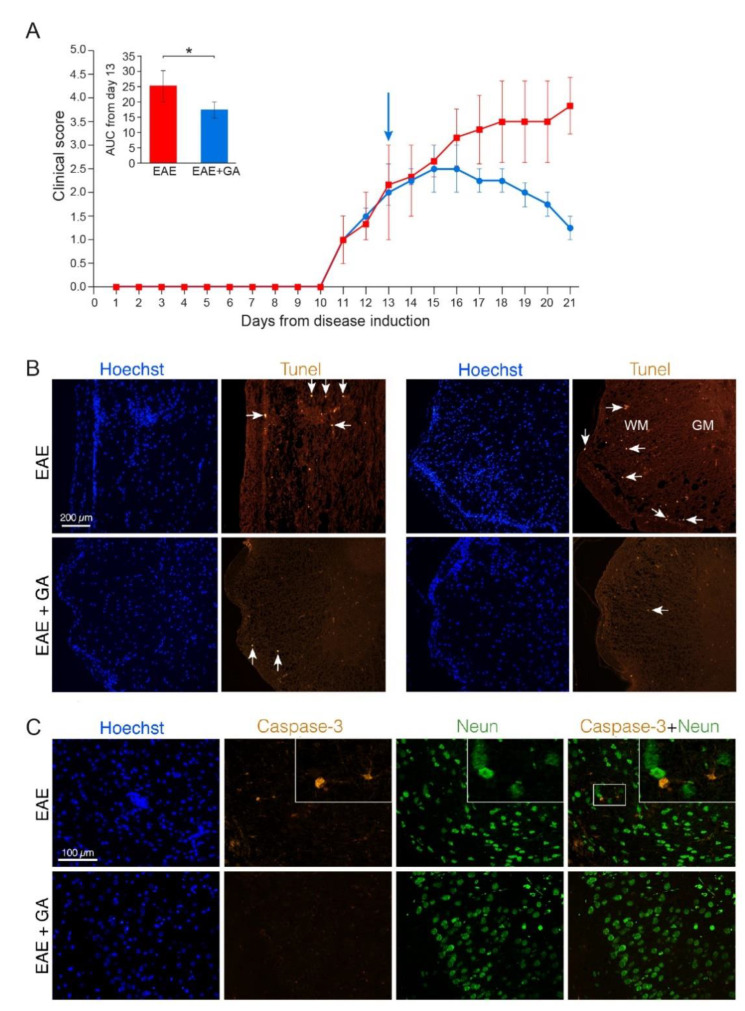
Apoptotic cell death in EAE induced mice, 21 days after disease induction, and the effect of GA-treatment. (**A**) Clinical scores of MOG-induced mice and GA-treated mice, treatment initiated at day 13. Average daily clinical scores ± SEM are presented for five mice per group. Insert is the area under curve (AUC) for days 13–21 ± SEM. Arrow indicates initiation of GA-injection period. * *p* < 0.05. (**B**) Tunel labeling in the spinal cord. Examples from two EAE-untreated and two EAE+GA mice are shown. In the white matter (WM), but not in gray matter (GM) of EAE-untreated mice, Tunel positive cells are detected in sites of cell infiltrations (indicated by Hoechst staining), whereas in GA-treated mice, less inflammation and less Tunel positive cells are seen. Arrows indicate examples of positive Tunel staining. (**C**) Caspase-3 staining in the brain. Caspase-3 positive cells, negative for NeuN are detected in the cortex of EAE-untreated mice. In GA-treated mice less Caspase-3 positive cells are seen.

**Figure 2:**
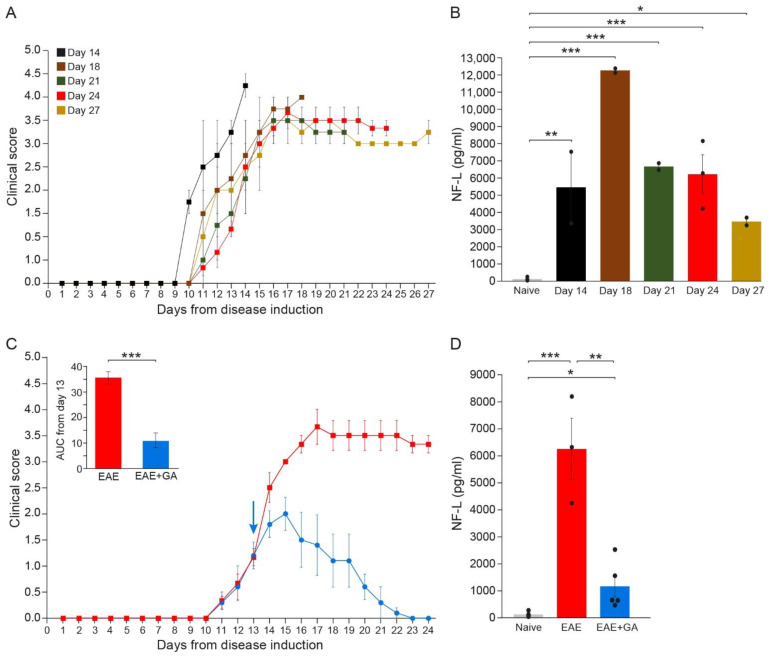
The kinetics of NFL leakage in sera of EAE-mice along the disease duration and the effect of GA-treatment. (**A**) Clinical daily scores of the EAE groups tested at different time-points after disease induction. (**B**) NFL concentration (pg/mL) in the serum of EAE-mice along the disease duration. (**C**) Clinical daily scores of EAE-untreated mice and the effect of GA daily suppression treatment, initiated at day 13. The insert is the area under curve (AUC) for days 13–24 ± SEM. Arrow indicates initiation of GA treatment. (**D**) NFL concentration (pg/mL) in the serum of EAE-untreated and GA-treated mice, 24 days after disease induction. The average values ± SEM for 2–5 mice (measured in duplicates for each mouse) are demonstrated. Values of individual mice are depicted by dots. NFL concentrations determined by single molecule array (Simoa). * *p* < 0.05, ** *p* < 0.01, ***, *p* < 0.001.

Damage manifestations in EAE, and the consequences of GA-treatment on these manifestations were studied using the MOG 35–55 peptide-induced EAE model, in which chronic (non-remitting) clinical symptoms are typically seen 10–11 days after disease induction, increasing in severity and reaching an average score of 3–4 (hind body or complete paralysis) by day 15–17. GA-treatment, applied as a suppression therapeutic regimen, initiated 13 days after disease induction to mice with apparent clinical symptoms (injected daily till the end of each experiment), resulted in a substantial decline of the clinical score. Layouts of the daily clinical scores, as well as the area under curve (AUC) for the treatment days of the mice used in this study, EAE-induced untreated (EAE) versus EAE-induced treated by GA (EAE+GA), are presented for each experiment (Figures 1, 2 and 4).

### 2.1. Apoptosis

To study programed cell death we stained for both Tune, a marker of DNA fragmentation, and Caspase-3, a crucial mediator of apoptosis. Representative depictions from EAE-untreated and EAE+GA mice (five mice examined per group, 21 days after disease induction) are presented in Figure 1, along with their averaged daily clinical scores and AUC (A). Positive Tunel labeling was obtained in EAE-untreated mice in sites of cell infiltrations (indicated by Hoechst staining), in the white matter regions, and not in the gray matter where the neuronal cell bodies are located (shown in spinal cord sections from two untreated mice in Figure 1B, upper row). A similar pattern was found using Caspase-3 staining, namely apoptotic cells in infiltration sites in the white matter, and not in the gray matter. When cortical gray matter brain sections were double stained for Caspase-3 and the neuronal marker NeuN, only a few Caspase-3 positive cells could be detected, and these cells were negative for the neuronal marker NeuN (Figure 1C, upper row). Quantification of Caspase-3 and NeuN in the cortex of EAE mice revealed 28 caspase-positive cells in an area of 4 mm^2^, from which only two cells (7%) were positive for NeuN. These findings imply that in MOG-induced EAE apoptotic programed cell death occurs mainly in infiltrating immune cells or other non-neuronal cell-populations. Accordingly, the neuronal loss seen in the chronic MOG-induced model cannot be explained by apoptotic neuronal cell death.

In spinal cords and brains of GA-treated mice, less cell infiltrations and less Tunel and Caspase-3 positive cells (6 caspase positive cells, negative for NeuN, in an area of 4 mm^2^) were detected (Figure 1B,C, lower rows). Since the cell death depicted by these markers in EAE-mice is not attributed to neuronal cell death, the reduced apoptotic consequence obtained by GA-treatment may be due to its anti-inflammatory activity.

### 2.2. NFL Leakage

Axonal and neuronal CNS damages were assessed by measuring the NFL leakage into the periphery (the serum) of MOG-induced mice along the disease duration. The daily clinical scores of the mice, tested at the different time-points, are presented in Figure 2A. Serum NFL concentrations were determined in duplicates, 2–5 mice in each time point, by the ultra-sensitive immunoassay, single molecule array (Simoa).

As depicted in Figure 2B, while in healthy mice, NFL levels were on average 118 ± 39 pg/mL (close to the minimal detection level), substantial NFL elevation was detected in all EAE-inflicted untreated mice (significant differences between naïve and EAE for all times when each time-point was compared to normal). Thus, four days after the appearance of clinical manifestations (14 days from disease induction) an average amount of 5471 ± 2097 pg/mL was already detected in mice manifesting aggressive (stage 4) disease (*p* = 0.003), reaching a peak of 12,300 ± 122 pg/mL by day 18 (*p* < 0.001), indicative of the extensive axonal damage occurring in the MOG model as a primary pathological characteristic. Still, it should be noted that the mice tested at day 14 manifested a somewhat more aggressive disease, reaching a clinical score of 4 (complete paralysis), while the scores of most of the mice at this time-point were 2–3. Thereafter, NFL concentration declined, reaching 6700 ± 201 pg/mL (*p* < 0.001) and 6244 ± 1140 pg/mL (*p* < 0.001) by days 21 and 24, respectively, and 3481 ± 224 pg/mL by day 27 (*p* = 0.046). Yet, when all groups were jointly compared, the differences between day 27 and naïve did not cross the significance level (*p* = 0.08). Notably, in two mice which showed a weak disease presentation (maximal clinical score 2 and 2.5 at day 17, and subsequently spontaneous recovery), the NFL level was 1794 ± 6 pg/mL, half of that found at the same time-point (day 27) in mice that did not recover. In mice that were induced with EAE but did not show any clinical manifestations, as well as in mice injected with CFA alone (without MOG), NFL serum concentrations were under 120 pg/mL, similar to those of naïve controls. These findings support NFL as a biomarker reflecting disease activity and the underlying CNS pathology.

In EAE-mice treated with GA (Figure 2C), NFL levels were drastically lower than in EAE-untreated mice (Figure 2D). An average concentration of 1162 ± 390 pg/mL was measured in five GA-treated mice compared to 6244 ± 1140 pg/mL in untreated mice tested at the same time-point (day 24), a significant decrease of 81% (*p* = 0.008). Yet, NFL levels in GA-treated mice were still higher than in naïve mice (*p* = 0.02).

**Figure 3:**
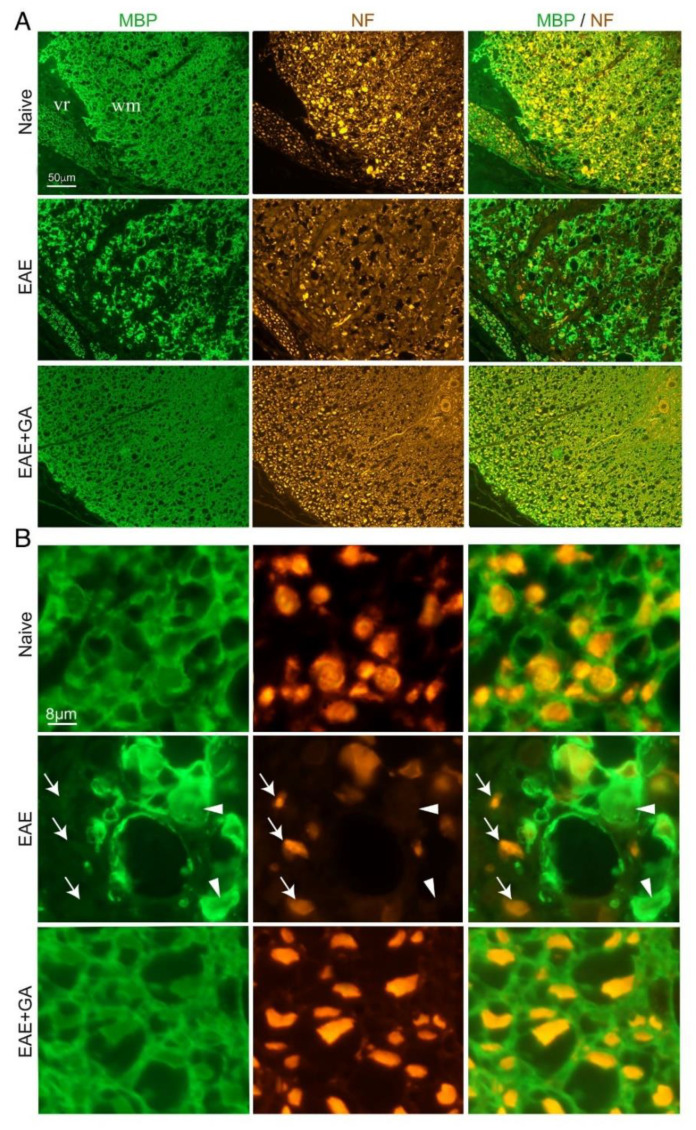
Immunohistological characteristics in spinal cords of EAE-induced mice, 24 days after disease induction and the effect of GA (suppression treatment). Myelin is visualized by anti-myelin basic protein (MBP) and axons by anti-neurofilament (NF). (**A**) Ventral lateral spinal cord area. (**B**) Inserts with higher magnifications. In EAE-untreated mice, sites of demyelination (arrows) and axonal loss (arrowheads) are revealed, whereas in GA-treated mice, damage to myelin and axons is less apparent. WM- CNS white matter, vr- ventral roots in the periphery.

To find out if NFL leakage reflects actual CNS damage, we performed immunohistological analyses in the spinal cord of EAE-induced mice, untreated and GA-treated (24 days from disease induction), as well as of naïve controls. Representative images (from three mice inspected), in which myelin is visualized by MBP-antibodies and axons by NF-antibodies, are presented in Figure 3. In the white matter of naïve mice, multiple axonal fibers are seen, depicted as round puncta, and encircled by myelin rings. In contrast, in spinal cords of EAE-untreated mice, widespread areas of myelin damage and loss are evident. Extensive axonal deformation and loss are evident in sites of demyelination, and remaining NF puncta appeared “naked” devoid of myelin envelops. Multiple “empty” myelin envelops without NF expressing fibers are also seen. Thus, in addition to the characteristic demyelination, severe axonal pathology and tissue damage are prevalent in this disease, facilitating the leakage of NFL into the periphery. Notably, loss of MBP and NF staining in EAE is apparent in the CNS, but not in the periphery, as depicted in Figure 3 by the intact spinal ventral roots, confirming that NFL detected in the sera of diseased mice originates in the CNS.

In spinal cords of GA-treated mice, axonal damage/loss and demyelination are considerably less prevalent, and tissue formation looks similar to that of naïve mice. This decrease in CNS tissue damage histologically seen in the CNS, together with the reduced NFL leakage obtained in the periphery of GA-treated mice, supports an actual protective consequence of GA-treatment.

### 2.3. Glutamate Excess

**Figure 4:**
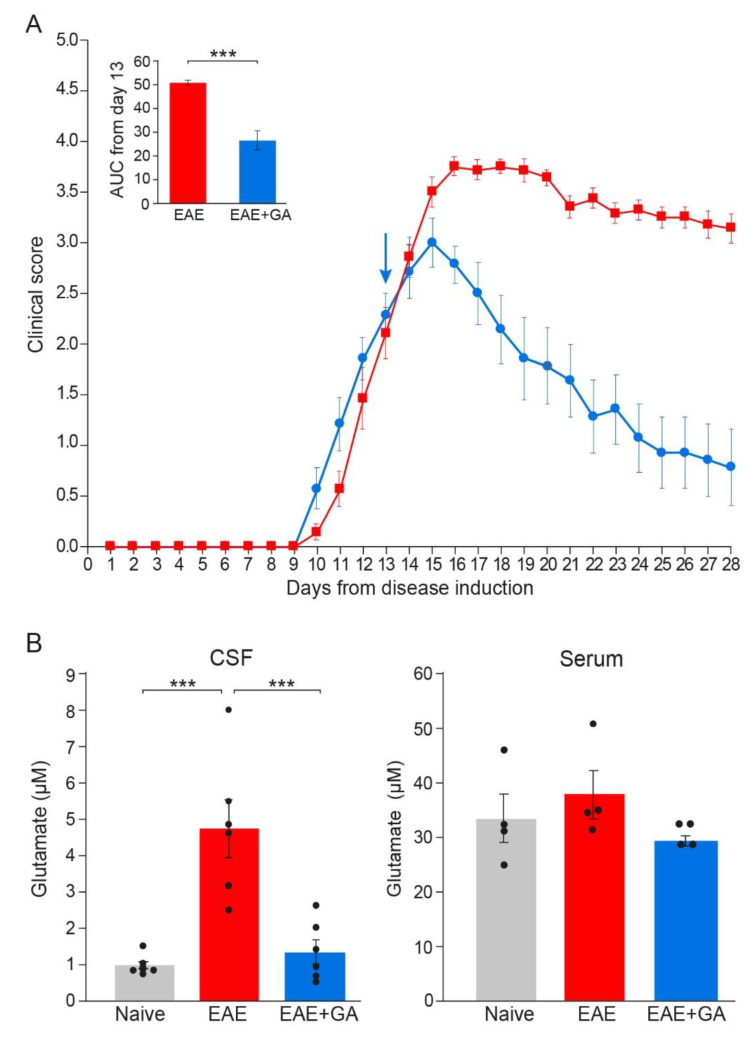
Glutamate levels in EAE-mice and the effect of GA-treatment. (**A**) Clinical scores of EAE mice and the effect of GA daily suppression treatment, initiated at day 13. The average daily clinical scores ± SEM are presented for six mice per group. The insert is the area under curve (AUC) for days 13–28 ± SEM. Arrow indicates initiation of GA treatment. (**B**) Glutamate concentrations (µM) in the cerebrospinal fluid (CSF, left), and in the periphery (serum right) of naïve, EAE-untreated, and EAE+GA mice, determined 28 days after disease induction, by mass spectrometry. The average concentration ± SEM of six mice per group for the CNS and four mice per group for the serum is shown. Values of individual mice are depicted by dots. *** *p* < 0.005.

To assess glutamate levels in the CNS of EAE-mice, we extracted cerebrospinal fluid (CSF) from the cisterna magna of naïve, EAE-untreated and EAE+GA mice, six mice per group, 28 days after disease induction (the daily clinical scores and AUC are depicted in Figure 4A). Extracellular glutamate levels were determined in the CSF and the serum of individual mice by microanalysis chromatographic system and mass spectrometry. Chromatography data and plots are described in the supplementary material.

Glutamate concentrations in the CSF and the serum are shown in Figure 4B. Serum, glutamate levels of all the mice tested were in the range of 25–50 µM, and the changes in EAE-mice versus naïve controls were minor and insignificant. In contrast, in the CSF, where normal glutamate levels were on average 0.93 ± 0.25 µM, an average concentration of 4.73 ± 1.95 µM was detected in EAE-untreated mice, a 5.1-fold elevation from naïve mice (*p* = 0.0002). This in situ elevation of glutamate level in EAE mice implies that glutamate excess may be a factor in the damage inflicted on the CNS cell populations during this disease. It should be noted, that significant elevation in glutamate was detected long after disease induction (day 28), while two weeks after EAE induction, glutamate concentration in the CSF of EAE-mice was only 1.57 ± 0.45 µM, an insignificant change from naïve mice.

GA treatment, applied as a suppression regimen to mice with clinical scores of 2–2.5, resulted in a lower CSF glutamate level, an average of 1.30 ± 0.82 µM, namely a decrease of 72% from untreated mice, which is a significant difference from EAE-untreated (*p* = 0.0006), but an insignificant difference from naïve controls (*p* = 0.866). This novel effect, namely elimination of glutamate excess in the CNS, may play a role in the protective consequence of GA-treatment on CNS tissue damage.

## 3. Discussion

MS is increasingly acknowledged as a neurodegenerative disease triggered by an inflammatory attack on the CNS [2,3,4,5,6,7,8]. While the inflammatory process is generally recognized, the processes that mediate neurodegeneration are less clear. In this study, we investigated neurodegenerative manifestations in the MS animal model, chronic MOG 35–55 peptide induced EAE, in which axonal and neuronal pathologies are prevalent [9,10,11]. Neuronal pathology particularly is manifested in this model by swelling of cell bodies and nucleus margination, as well as by progressive neuronal loss [9,10]. Neurodegeneration comprises a diverse pool of neuronal cell death, apoptosis, necrosis, and hypoxia [8]. Indeed, apoptosis of neurons have been described in MS patients [42,43,44] and in various EAE models [44,45,46,47]. In the current study, using two markers—Tunel and Caspase-3—to search for apoptosis in spinal cord and brain sections of MOG-induced mice, we detected apoptotic cell death, mainly in inflammation sites in white matter regions, and not in neuronal cell bodies in the gray matter. Furthermore, the few apoptotic cells found in gray matter regions were negative for the neuronal marker NeuN. Our findings imply that apoptotic programmed cell death occurs mainly in infiltrating immune cells or other non-neuronal cell-populations, and thus cannot account for the neuronal loss and the extent of tissue damage prevailing in this disease.

Another mechanism that can lead to neuronal loss is glutamate toxicity. This key excitatory neurotransmitter induces when in excess excitotoxic cell death. We demonstrate here that in the CSF of MOG-induced mice there is a 5.1-fold elevation in glutamate concentration compared to naïve mice, indicating that glutamate excess might be a factor in the neurodegeneration prevailing in this model. This elevation can be attributed to excessive glutamate production by invading immune cells and activated CNS resident astrocyte/microglia, as well as to the reported abnormalities in glutamate degrading enzymes, transporters, receptors, and signaling [27,28,29,30,31]. It should be noted that significant glutamate elevation was detected only after an extended period from disease induction, on day 28. Therefore, excessive glutamate cannot account for early neuroaxonal damage. These results are in accordance with our previous study demonstrating significant neuronal loss at days 36 and 50 in the MOG-induced EAE model [9].

Extensive neuroaxonal damage was indicated by substantial NFL elevation in the sera of EAE-induced mice, exceeding 100-fold from the basal concentration in healthy mice, 18 days after disease induction. NFL, a constituent of the axonal cytoskeleton, is released from the injured fibers, leaks to the blood in the periphery, and may thus be used as an indicator for neurodegeneration [17,18,19,20,21,22]. However, the limited knowledge of NFL dynamics in the sera, as well as its connection to the ongoing tissue damage and disease activity presents a barrier for its usage as a standard biomarker. Here, by performing detailed kinetics of NFL leakage in EAE mice along time, we show that a prominent elevation occurs subsequent to clinical symptoms appearance, reaching a peak already 18 days after disease induction. Notably, NFL concentration in mice which showed a weak disease manifestation (maximal clinical score of 2–2.5 and thereafter recovery) is half of that found in mice enduring severe chronic disease (score of 3 with no recovery) at the same time-point. Furthermore, histological analyses revealed severe axonal deformation and loss in the CNS, but not in the periphery of EAE-induced mice, confirming that the NFL detected in the sera of diseased mice originates in the CNS. These combined results support using NFL as a sensitive and reliable biomarker, reflecting disease activity and the underlying CNS pathology.

These findings further substantiate the extensive neuroaxonal damage, occurring particularly in the MOG model, as a primary pathological characteristic, which is in accord with our previous immunohistochemistry, electron microscopy and MRI imaging analyses [9,10,11,12]. It is worth noting that in mice induced by the relapsing-remitting PLP-EAE model, we detected lower NFL levels (an average of 9533 pg/mL, compared to 12,300 pg/mL in the MOG model at the same time-point). Furthermore, in an Alzheimer model (5×FAD), we detected markedly lower NFL concentrations (an average of 758 pg/mL. Similar NFL levels were previously reported in MOG-induced EAE at a single time-point (day 16–17) [23], and in the 5×FAD Alzheimer model [48]. Notably, we found that NFL levels decline with time, but remain high (57 and 53-fold of the normal level, 21 and 24 days from disease induction, respectively, and 30-fold by day 27), even though the clinical scores are stable (grade 3–3.5 till day 27). This agrees with previous studies demonstrating that in the MOG-EAE model axonal loss coincides with the initial clinical signs and does not recover over time [49,50].

GA, given as a therapeutic treatment to mice with apparent disease symptoms, diminished the above pathological manifestations, namely, clinical score, apoptotic cell death, glutamate excess, histological tissue damage, and NFL leakage. The apoptotic cell death depicted by Tunel and Caspase-3 in untreated mice is attributed mainly to infiltrating immune cells and not to neurons. Thus, the decrease obtained following GA treatment should be referred to its renowned anti-inflammatory activity, such as lowering immune cell infiltration [9,35,51]. The decrease in glutamate concentration in the CSF may also be linked to the ability of GA to reduce immune cells infiltration, as well as its ability to reduce microglial and astrocyte activation [10,35,52]. It has been reported that GA affects glutamate transmission alterations in the nucleus striatum of EAE mice by attenuating microglial activation [53]. The novel effect found in the current study, namely elimination of interstitial glutamate excess, may play a role in reducing excitotoxic neuronal and oligodendrocyte cell death, thus reducing CNS tissue damage.

The neuroprotective effect of GA is also evident in this study by the significant reduction in NFL concentration in sera of GA-treated mice (a decrease of 81% compared to untreated mice), indicative of milder neuroaxonal injury. This was also confirmed by immunohistological analyses, in which considerably less axonal deformation and loss were detected in the spinal cord of GA-treated mice. NFL levels in GA-treated mice were still higher compared to naïve controls (significant differences, *p* = 0.02). This could result from a certain neuronal damage persisting following treatment. It should be noted that GA was applied as a therapeutic regimen, after disease outburst (at day 13), when neuroaxonal damage was already manifested in this model [11,49,50]. Reduced serum NFL levels following GA treatment, associated with disease activity and therapy response, was also reported in MS patients [54]. The combined findings presented here substantiate the notion that GA treatment augments neuroprotective processes and counteracts the neurodegenerative disease pathologies.

## 4. Materials and Methods

### 4.1. Mice

C57BL/6 mice were purchased from Envigo (Jerusalem, Israel). Female mice, 8–12 weeks old, were kept in a specific pathogen free (SPF) environment. All experiments were approved by the Institutional Animal Care and Use Committee of the Weizmann Institute and were performed according to their guidelines and regulations.

### 4.2. EAE Induction and Evaluation

EAE was induced by the peptide encompassing amino acids 35–55 of myelin oligodendrocyte glycoprotein (MOG), synthesized by Genscript (Piscataway, NJ, USA). Mice (5–15 animals per experiment) were injected subcutaneously with 100 μL emulsion containing 200 μg of the peptide in incomplete Freund’s adjuvant enriched with 3.3 mg/mL heat-inactivated Mycobacterium Tuberculosis (Sigma-Aldrich, St. Louis, MO, USA). Pertussis toxin (Sigma-Aldrich), 150 ng/mouse, was injected intraperitoneally immediately after the encephalitogenic injection and 48 h later. The mice were examined daily, and EAE was scored as follows: 1—loss of tail tonicity, 2—hind limb weakness or partial paralysis, 3—hind leg paralysis, 3.5—hind leg complete paralysis with hind body paresis, 4—hind and foreleg paralysis, 5—death.

### 4.3. Glatiramer Acetate (GA, Copaxone, Copolymer 1)

GA containing four amino acids, L-alanine, L-glutamate, L-lysine, and L-tyrosine, was obtained from Teva Pharmaceutical Industries (Petah Tiqva, Israel). GA-treatment was applied by consecutive daily subcutaneous injections, 2 mg per mouse in 0.1 mL phosphate buffered saline (PBS), as a suppression treatment starting 13 days after disease induction in mice with apparent clinical symptoms (injected daily till the end of each experiment). These regimens and treatment dose of GA were found effective in our previous studies in the EAE system [9,10,11,12,34,38]. Mice not treated with GA were injected with PBS alone. The layouts of the experimental systems and GA-treatment schedules are shown in Figure 1, Figure 2 and Figure 4.

### 4.4. Immunohistochemistry

Animals were euthanized by an overdose of anesthesia. Brains and vertebral columns were dissected and fixed in paraformaldehyde (2.5% for 48 h, and 1% for 2–4 days). Vertebral columns were decalcified with 12.5% EDTA (pH 7.2) followed by spinal cord segments dissection. Brains and spinal cords were then paraffin embedded, and sectioned coronally (4 μm) by a microtome. For staining, paraffin sections were deparaffinized and rehydrated. Antigen retrieval was performed in 10 mM citric acid pH6 for 10 min in a microwave, to break protein crosslinks and unmask the antigens. After pre-incubation with 20% normal horse serum and 0.2% Triton X-100, sections were incubated with primary antibodies at RT for 24 h. The following antibodies were used: rat anti-myelin basic protein (MBP, 1:50, Abcam, Cambridge, UK), rabbit anti-neurofilament light, medium, and heavy protein (NF, 1:50, Novus, Littleton, CO, USA), rabbit anti-caspase-3 (1:50, Cell Signaling Technology, Denver, MA, USA), mouse anti-neuronal-specific nuclear protein (NeuN) (1:300; Millipore, Burlington, MA, USA). The second antibody step was performed by labeling with specie specific cy2, cy3 or cy5 conjugated antibodies (1:100, Jackson ImmunoResearch, West Grove, PA, USA) for 30–40 min. In some cases, the signal was enhanced by incubation with biotinylated secondary antibodies for 90 min, followed by cy2 or cy5 conjugated streptavidin (1:100, Jackson ImmunoResearch). Sections were counterstained with Hoechst 33,258 (Molecular Probes, Eugene, OR,USA) for nuclear labeling. Tunel detection was performed using an apoptag kit (Millipore).

### 4.5. Neurofilament Light (NFL) Measurement

Mice were anaesthetized and blood samples were collected. Serum was frozen and stored at −80 °C until analysis. Sera were initially diluted 1:1000. When an obtained concentration was higher than 500 pg/mL, an additional dilution of 1:5000 was further tested. NFL concentrations were measured in duplicates by a single molecule array (Simoa) assay (Quanterix, Boston, MA, USA) and by a commercial kit (NF-light Advantage Kit, UmanDiagnostics Umea, Sweden), using a bead-conjugated immunocomplex. The immunocomplex was applied to a multi-well array designed to enable imaging of every single bead. The average number of enzymes per bead (AEB) of each sample was interpolated onto the calibrator curve constructed by AEB measurements on bovine NFL (UmanDiagnostics), serially diluted in an assay diluent. Samples were analyzed using one batch of reagents. Animal treatment information was blinded to the investigator performing the analysis.

### 4.6. Cerebrospinal Fluid (CSF) Collection

Mice were anaesthetized with ketamine (200 mg/kg) and xylazine (10 mg/kg). A midline incision was made between the mice ears until the base of the skull was exposed and the connective tissue was pulled apart from the area above the cisterna magna. Once the dura over the cisterna magna was exposed, the membrane over the cisterna magna was cleaned using a dry cotton bud. A capillary with a sharpened point was used to delicately pierce the cisterna magna membrane at a 45° angle and transparent CSF fluid was drained. CSF samples were immediately snap frozen and stored at −80 °C for later analyses.

### 4.7. Glutamate Analysis

Glutamate concentrations were determined in the CSF and the serum of individual mice by microanalysis using a chromatographic system and mass spectrometry in the Department of Life Sciences Core Facilities (the Chemical Services Division of the Weizmann Institute). Briefly, samples were diluted 1:10 in 0.1% formic acid. A glutamate derivatization procedure was performed using 6-aminoquimolyl-N-hydroxysuccinimidyl carbomate (AQC) reagent. A 10-μL aliquot of the samples or standard solution and 70 μL of 0.15 M sodium borate solution, pH 8.8, were derivatized with 20 μL of AQC in acetonitrile (2.7 mg/mL) by heating at 55 °C for 10 min. The reaction mixtures were cooled and placed in nanofilter vials for liquid chromatography–mass spectrometry (LC-MS). For mass spectrometry, argon was used as the collision gas. The capillary voltage was set to 3.00 kV, cone voltage 25 V, source offset 30 V, source temperature 150 °C, desolvation temperature 650 °C. Glutamate was detected using selected reaction monitoring (SRM) and retention times. Chromatography methods, data, and plots are further described in the supplementary material.

### 4.8. Statistical Analyses

The area under curve (AUC) values of individual mice were compared between the untreated and GA-treated mice, using a t-test for unequal variances. Glutamate levels of individual mice were compared between naïve, untreated, and GA-treated mice, using a one-way ANOVA, followed by Tukey’s post-hoc test. NFL levels of individual mice were compared between groups using a one-way ANOVA, followed either by Tukey’s post-hoc test to compare all the groups or by Dunnett’s test to compare all days vs. normal. To compare between naïve, untreated, and GA-treated mice, data was log-transformed because of the mean-variance correlation. Statistical significance is indicated by an * for *p* ≤ 0.05, ** for *p* ≤ 0.01, *** for *p* ≤ 0.001.

## Data Availability

The data presented in this study are available in the Results, Materials and Methods, and supplementary sections. Additional data will be available on request.

## Supplementary Materials

The following are available online at https://www.mdpi.com/article/10.3390/ijms222413419/s1.
