# Supplementary material for: Neuroprotective Effect of Glatiramer Acetate on Neurofilament Light Chain Leakage and Glutamate Excess in an Animal Model of Multiple Sclerosis
Source: Int J Mol Sci. 2021 Dec 14;22(24):13419. doi: 10.3390/ijms222413419 (PMC8707261; doi:10.3390/ijms222413419)
Supplement: Supplementary file 1 [file ijms-22-13419-s001.zip › ijms-1470566 supp.pdf]

Glutamate analysis

Derivatization of Glutamate

Samples were diluted 1:10 in 0.1% formic acid. Derivatization procedure was performed using AQC reagent synthesized as described [1]. Briefly, a 10uL aliquot of the sample or standard solution (with the internal standards added) and 70uL of 0.15 M sodium borate solution, pH 8.8 were derivatized with 20 uL of AQC in acetonitrile (2.7mg/mL) by heating at 55°C for 10 min. The reaction mixtures were cooled and placed in nanofilter vials (Thomson, 0.2um PES) for LC-MS.

LCMS analysis

The LC-MS/MS instrument consisted of Acquity I-class UPLC system (Waters) and Xevo TQ-S triple quadrupole mass spectrometer (Waters) equipped with an electrospray ion source and operated in positive ion mode was used for analysis. MassLynx and TargetLynx software (v.4.1, Waters) were applied for the acquisition and analysis of data. Chromatographic separation was done on a 150 x 2.1-mm i.d. 1.8-µm UPLC HSS T3 column equipped with 50 x 2.1-mm i.d., 1.8-µm UPLC HSS T3 pre-column (both Waters Acquity) with 0.1% formic acid as mobile phase A and 0.1% formic acid in acetonitrile as B at a flow rate of 0.6 ml/min and column temperature 45°C. A gradient was as follows: 0.5 min the column was hold at 4%B, then linear increase to 10%B in 2 min, then to 28%B in 2.5 min, and to 95%B in 0.1 min. Just after back to 0%B during 1.1 min, and equilibration at 4%B for 1.3 minutes. Samples kept at 4°C were automatically injected in a volume of 1 µl.

For mass spectrometry argon was used as the collision gas with flow 0.10 ml/min. The capillary voltage was set to 3.00 kV, cone voltage 25V, source offset 30V, source temperature 150°C, desolvation temperature 650°C, desolvation gas flow 800 L/hr, cone gas flow 150 L/hr. Glutamate was detected using selected reaction monitoring (SRM) and retention time. The MRM used was 318.1>171.2 and the RT was 2.5min (The concentrations based on standard curves were calculated using TargetLynx (Waters). Calibration plot is presented below

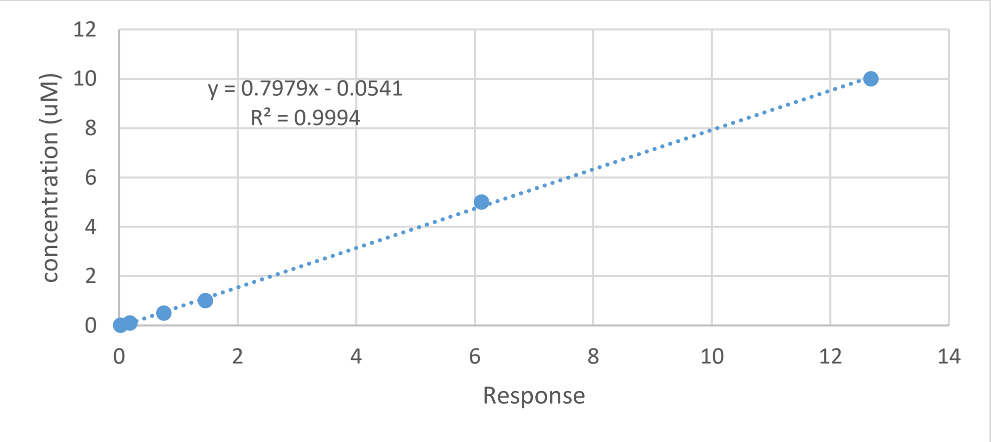

Reference

1. Cohen, S. A.; Michaud, D. P. Synthesis of a fluorescent derivatizing reagent,6-aminoquinolyl-N-hydroxysuccinimidyl carbamate, and its application for the analysis of hydrolysate amino acids via high-performance liquid chromatography. *Anal. Biochem.* **1993**, *211*, 279-287

**Sample**	**Serum glutamate** µm	Sample description
1	45.7	Naïve serum
2	32.0	
3	30.7	
4	24.6	
5	50.5	EAE serum
6	34.7	
7	31.0	
8	34.6	
9	27.5	GA serum
10	28.9	
11	28.0	
12	31.7	
13	0.85	Naive CNS
14	1.43	
15	0.83	
16	0.81	
17	0.95	
18	0.74	
19	5.45	EAE CNS
20	4.56	
21	3.12	
22	7.99	
23	4.82	
24	2.44	
25	2.57	GA CNS
26	1.34	
27	0.62	
28	1.96	
29	0.44	
30	0.87	
